# Impact of retrieved lymph node count on short-term complications in patients with gastric cancer

**DOI:** 10.1186/s12957-020-02000-9

**Published:** 2020-08-24

**Authors:** Feng Sun, Song Liu, Peng Song, Chen Zhang, Zhijian Liu, Wenxian Guan, Meng Wang

**Affiliations:** Department of Gastrointestinal Surgery, Nanjing Drum Tower Hospital, The Affiliated Hospital of Nanjing University Medical School, Nanjing, China

**Keywords:** Retrieved lymph nodes, Postoperative complications, Gastric cancer

## Abstract

**Background:**

It is well established that retrieved lymph node (RLN) counts were positively correlated with better overall survival in gastric cancer (GC). But little is known about the relationship between RLN count and short-term complications after radical surgery.

**Methods:**

A total of 1487 consecutive GC patients between January 2016 and December 2018 at Nanjing Drum Tower Hospital were retrospectively analyzed. Univariate analyses were performed to elucidate the association between RLN count and postoperative complications. We further identified clinical factors that might affect the RLN count.

**Results:**

Among all of the patients, postoperative complications occurred in 435 (29.3%) patients. The mean RLN count was 25.1, and 864 (58.1%) patients were diagnosed with lymph node metastasis. Univariate analyses showed no significant difference between RLN count and postoperative complications (both overall and stratified by CDC grade). Univariate and multivariate analyses further revealed that type of resection, tumor invasion, and lymph node metastasis were associated with RLN count.

**Conclusions:**

The current study demonstrated that RLN count was not associated with postoperative short-term complications following gastrectomy of GC, which provided a rationale for the determination of a proper RLN count of curative gastrectomy.

## Background

There are approximately one million new cases of gastric cancer (GC) each year worldwide, and half of them occur in Eastern Asia, including China, Japan, and South Korea [1]. Despite advances in early screening and comprehensive treatment of GC, it remains the third most common cause of cancer-related death in the world [2]. For advanced GC, a consensus has been reached of radical gastrectomy with D2 lymphadenectomy [3]. However, there is still controversy over the number of retrieved lymph nodes (RLNs) for accurate pathological staging.

Several studies have reported that RLN count was positively correlated with better overall survival in GC, even in lymph node-negative GC [4–7]. An RLN count of ≥ 16 has been recommended by the 8th edition TNM classification for GC to guarantee the accurate pN stage [8]. Moreover, Okajima et al. suggested an optimal RLN count of ≥ 25 for nodal staging [9]. Recently, by stratum analysis of 7620 patients, Deng et al. proposed an optimal RLN count of ≥ 16 for lymph node-negative GC and > 30 for lymph node-positive GC [10]. These above studies are all conducted by comparing the RLN count with long-term survival. But little is known about the relationship between the RLN count and short-term complications after radical surgery.

Postoperative complications of GC pose a significant impact on the length of postoperative stay and hospital charges, which further affect the quality of life [11]. Therefore, investigating the relationship between RLN count and postoperative short-term complications would provide more comprehensive evidence for selecting the appropriate RLN count.

## Methods

### Patients

A total of 1487 consecutive GC patients between January 2016 and December 2018 at Nanjing Drum Tower Hospital were retrospectively reviewed. All patients underwent curative (R0) gastrectomy and were histologically confirmed. The exclusion criteria were as follows: (1) multivisceral resection, (2) patients accepting preoperative radiotherapy or chemotherapy, (3) patients with previous stomach surgery, and (4) patients with incomplete clinical data. This study was approved by the Ethics Committee of Nanjing Drum Tower Hospital.

### Data collection

Data for preoperative characteristics, intraoperative index, and postoperative features were extracted. Preoperative characteristics included age, gender, body mass index (BMI), comorbidities, and laboratory data. The intraoperative index involved the American Society of Anesthesiologists (ASA) grade, surgical approach, type of resection, operation time, and blood loss. Postoperative features included depth of tumor invasion, tumor site, retrieved lymph node count, lymph node metastasis, lymph node ratio (LNR), log odds of positive lymph nodes (LODDS), pTNM stage, Lauren subtype, short-term complications, postoperative stay, and total hospital charges. LNR was defined as the ratio of positive to retrieved lymph nodes. LODDS was calculated by log [(positive lymph nodes + 0.5)/(total lymph nodes − positive lymph nodes + 0.5)] [12]. The postoperative short-term complications occurring in the hospital or within 30 days were collected. All complications were evaluated according to the Clavien-Dindo classification system [13].

### Statistical analysis

Statistical analyses were conducted by SPSS 19.0 (Chicago, IL, USA). Continuous variables were shown as means ± SD. Student’s *t* test was applied for normally distributed data; Mann-Whitney *U* test was applied for non-normally distributed data. Categorical variable data were presented as numbers and analyzed using the chi-squared test or the Fisher exact test. Univariate and multivariate analyses were performed to analyze the risk factors associated with the postoperative complications or retrieved lymph node count. The optimal cutoff values of LNR and LODDS were determined by receiver operating characteristic (ROC) analysis. All statistical tests were conducted two-sided, and statistical differences were termed as *P* value < 0.05.

## Results

### Patient characteristics

The background characteristics of the patients enrolled in this study were presented in Table 1. There were 1487 GC patients in all, including 1089 (73.2%) men and 398 (26.8%) women. The median age was 60 years with a range from 21 to 96 years. A total of 1411 (94.9%) patients underwent open gastrectomy while 76 (5.1%) underwent laparoscopic surgery. The type of resection was distal gastrectomy in 617 (41.5%) patients, proximal gastrectomy in 163 (11.0%), and total gastrectomy in 707 (47.5%). The mean operation time was 232 min, and the mean intraoperative blood loss was 221 ml. Pathological results were stage I/II/III/IV in 506/368/597/16 patients, respectively. The mean RLN count was 25.1 (range, 2–84), and 864 (58.1%) patients were tested with lymph node metastasis. Overall, postoperative short-term complications occurred in 435 (29.3%) patients. The mean postoperative stay was 12 days, and the mean total hospital charges were 7.5 × 10^4^¥.

**Table 1:** Demographic and clinical features of patients

Characteristics	*N* = 1487
Age (years)	60.4 ± 17.3
Gender (*n*)	
Male	1089
Female	398
BMI (kg/m^2^)	23.0 ± 3.5
Preoperative comorbidities (*n*)	
Previous abdominal surgery	209
Diabetes mellitus	131
Hypertension	488
Preoperative laboratory data	
Serum albumin (g/L)	39.4 ± 3.3
CRP (g/L)	6.0 ± 12.4
ASA ≥ 3	884
Mode of surgical approach (*n*)	
Laparoscopic	76
Open	1411
Type of resection (*n*)	
Distal gastrectomy	617
Proximal gastrectomy	163
Total gastrectomy	707
Operation time (min)	232.3 ± 61.8
Blood loss (ml)	221.8 ± 204.5
Tumor invasion	
T1–2	631
T3–4	856
Tumor site	
Cardia/fundus	452
Body	381
Pylorus/antrum	654
RLN count	25.1 ± 9.1
Lymph node metastasis	
Positive	864
Negative	623
LNR	0.17 ± 0.24
LODDS	− 0.96 ± 0.75
pTNM stage I/II/III/IV	506/368/597/16
Lauren subtype	
Intestinal	620
Diffuse	428
Mixed	401
Unknown	38
Postoperative complications	
Positive	435
Negative	1052
Postoperative stay (days)	12.0 ± 8.1
Total hospital charges (10^4^¥)	7.5 ± 3.5

*BMI* body mass index, *CRP* C-reactive protein, *ASA* American Society of Anesthesiologists, *RLNs* retrieved lymph nodes, *LNR* lymph node ratio, *LODDS* log odds of positive lymph nodes

### Association between perioperative characteristics and postoperative complications

As presented in Table 2, univariate and multivariate analyses indicated that postoperative short-term complications were significantly correlated with age, gender, level of preoperative serum albumin, and operation time. Stratified analyses by type of resection revealed that complications occurred frequently in proximal gastrectomy compared with total gastrectomy, while there was no significant difference between distal gastrectomy and total gastrectomy. No significant association was observed between RLN count and overall postoperative complications.

**Table 2:** Univariate and multivariate analyses of characteristics associated with postoperative complications

Characteristics	Univariate			Multivariate		
	OR	95% CI	*P*	OR	95% CI	*P*
Age ≥ 70	1.581	1.232–2.029	< 0.001	1.578	1.219–2.044	0.001
Gender						
Male	0.765	0.597–0.979	0.033	0.710	0.551–0.916	0.008
Female						
BMI (kg/m^2^)	0.988	0.956–1.020	0.449			
Preoperative comorbidities						
Previous abdominal surgery	0.996	0.722–1.374	0.982			
Diabetes mellitus	1.156	0.787–1.700	0.460			
Hypertension	1.128	0.891–1.428	0.317			
Preoperative laboratory data						
Serum albumin < 35 g/L	1.660	1.162–2.372	0.005	1.544	1.068–2.232	0.021
CRP ≥ 10 g/L	1.315	0.892–1.938	0.167			
ASA ≥ 3	1.047	0.834–1.315	0.693			
Mode of surgical approach						
Laparoscopic	0.684	0.394–1.188	0.178			
Open						
Type of resection			0.067			0.025
Total gastrectomy		Reference			Reference	
Distal gastrectomy	1.183	0.932–1.503	0.167	1.242	0.972–1.588	0.083
Proximal gastrectomy	1.503	1.047–2.157	0.027	1.613	1.117–2.329	0.011
Operation time	1.002	1.001–1.004	0.009	1.003	1.001–1.005	0.002
Blood loss	1.000	1.000–1.001	0.094			
Tumor site			0.780			
Cardia/fundus		Reference				
Body	1.054	0.811–1.370	0.693			
Pylorus/antrum	0.947	0.716–1.252	0.701			
Tumor invasion (T3–4)	1.216	0.968–1.527	0.093			
RLNs	0.991	0.979–1.004	0.165			
Lymph node metastasis	1.044	0.832–1.310	0.707			
LNR > 0.05	1.213	0.969–1.517	0.091			
LODDS > − 1.1	1.219	0.975–1.525	0.083			
pTNM stage (≥ III)	1.036	0.826–1.300	0.757			
Lauren subtype			0.952			
Intestinal		Reference				
Diffuse	0.866	0.427–1.754	0.689			
Mixed	0.904	0.442–1.848	0.782			
Unknown	0.925	0.452–1.894	0.832			

### Impact of RLN count on postoperative complications

Of the 1487 patients, 435 (29.3%) developed complications: 74% (323 of 435) encountered a single complication, and 26% (112 of 435) encountered multiple complications. The details of patients with short-term complications based on the Clavien-Dindo classification are 15.5% for grade I, 9.2% for grade II, 4.0% for grade III, 0.3% for grade IV, and 0.2% for grade V. The rate of major complications (CDC grade ≥ III) was 4.5%. The median RLN count in this study was 24. So, we divided all patients into two groups based on the median RLN count. Univariate analyses showed no significant difference between RLN count and postoperative complications (both overall and stratified by CDC grade) (Table 3).

**Table 3:** Univariate analyses of postoperative complications associated with RLN count

Characteristics	All	RLN count		*P* value
		< 25	≥ 25	
Overall (*n*)	435	248	187	0.062
Grade I (*n*)	231	132	99	0.198
Fever > 37.5 °C	144	85	59	
Emesis	156	83	73	
Pain	30	18	12	
Abdominopelvic collection	1	1	0	
Pleural effusion	4	4	0	
Grade II (*n*)	137	78	59	0.366
Blood transfusions	60	38	22	
Early postoperative bowel obstruction	2	1	1	
Gastroparesis	25	14	11	
Liver function abnormalities	1	1	0	
Wound infection	8	5	3	
Pneumonia	27	15	12	
Intra-abdominal infections	20	12	8	
Urinary tract infection	4	0	4	
Enteritis	3	1	2	
Bacteremia	14	7	7	
Grade III (*n*)	59	32	27	0.878
Anastomotic leakage	23	14	9	
Lymphatic leakage	8	3	5	
Pancreatic fistula	2	0	2	
Biliary fistula	1	0	1	
Bleeding	8	5	3	
Abdominopelvic collection	1	1	0	
Pleural effusion	9	5	4	
Intra-abdominal abscess	2	1	1	
Wound disruption	3	3	0	
Delayed wound healing	4	3	1	
Gastroparesis	1	0	1	
Early postoperative bowel obstruction	1	0	1	
Splenic necrosis	1	0	1	
Grade IV (*n*)	5	4	1	0.452
Heart failure	1	1	0	
Kidney failure	1	1	0	
Brain infarction	1	0	1	
MODS	2	2	0	
Grade V (*n*)	3	2	1	1.000
Grade ≥ III (*n*)	67	38	29	0.562

*RLNs* retrieved lymph nodes, *MODS* multiple organ dysfunction syndrome

### Factors associated with RLN count

We further explored the potential factors associated with RLN count. Univariate analyses revealed that preoperative serum albumin, type of resection, tumor invasion, lymph node metastasis, and pTNM stage were associated with RLN count (*P* < 0.05; Table 4). Stratification by type of resection showed that RLN count in either distal gastrectomy or proximal gastrectomy was significantly lower than that in total gastrectomy. Multivariate analyses further indicated that type of resection, tumor invasion, and lymph node metastasis were still significantly associated with RLN count (*P* < 0.05; Table 4).

**Table 4:** Univariate and multivariate analyses of factors associated with RLN count ≥ 25

Characteristics	Univariate			Multivariate		
	OR	95% CI	*P*	OR	95% CI	*P*
Age ≥ 70	0.873	0.689–1.105	0.259			
Gender						
Male	1.043	0.828–1.312	0.723			
Female						
BMI (kg/m^2^)	0.972	0.944–1.002	0.064			
Preoperative comorbidities						
Previous abdominal surgery	1.030	0.768–1.380	0.844			
Diabetes mellitus	0.960	0.670–1.375	0.822			
Hypertension	0.852	0.685–1.059	0.148			
Preoperative laboratory data						
Serum albumin < 35 g/L	1.484	1.048–2.102	0.026			
CRP ≥ 10 g/L	1.195	0.827–1.726	0.343			
ASA ≥ 3	0.892	0.725–1.098	0.282			
Mode of surgical approach						
Laparoscopic	1.282	0.808–2.036	0.292			
Open						
Type of resection			< 0.001			< 0.001
Total gastrectomy		Reference			Reference	
Distal gastrectomy	0.649	0.522–0.807	< 0.001	0.716	0.572–0.896	0.004
Proximal gastrectomy	0.334	0.231–0.485	< 0.001	0.357	0.245–0.519	< 0.001
Operation time	1.001	1.000–1.003	0.086			
Blood loss	1.000	1.000–1.001	0.482			
Tumor site			0.304			
Cardia/fundus		Reference				
Body	0.903	0.709–1.148	0.404			
Pylorus/antrum	1.119	0.869–1.442	0.382			
Tumor invasion (T3–4)	1.613	1.310–1.987	< 0.001	1.299	1.010–1.670	0.042
Lymph node metastasis	1.585	1.286–1.952	< 0.001	1.304	1.018–1.669	0.035
pTNM stage (≥ III)	1.555	1.263–1.914	< 0.001			
Lauren subtype			0.082			
Intestinal		Reference				
Diffuse	1.040	0.536–2.019	0.908			
Mixed	1.388	0.709–2.716	0.339			
Unknown	1.328	0.677–2.603	0.409			

*BMI* body mass index, *CRP* C-reactive protein, *ASA* American Society of Anesthesiologists, *RLNs* retrieved lymph nodes, *OR* odds ratio, *CI* confidence interval

## Discussion

Nodal involvement significantly affected the prognosis of GC patients because it is the major root of tumor relapse after surgery [14, 15]. Thus, standardized lymph node dissection is the basic requirement for curative (R0) gastrectomy. Curative gastrectomy with D2 lymphadenectomy has been considered as the standard fashion for decades in Eastern Asia, especially in Japan [16, 17]. This procedure has been gradually accepted by Western countries in recent years [18, 19]. As for the RLN count, the 8th edition TNM classification for GC recommended dissecting at least 16 lymph nodes. Moreover, emerging evidence revealed the positive correlations between RLN count and overall survival of GC patients [4, 5, 20]. By comparing RLN count to survival time, Okajima et al. suggested an optimal RLN count of ≥ 25 [9]; Deng et al. proposed an optimal RLN count of ≥ 16 for lymph node-negative GC and > 30 for lymph node-positive GC by stratum analysis of 7620 patients [10]; Sano et al. reported that RLN count preferably achieved 30 or more by a multicenter study enrolling 25,411 patients [20]. Additionally, LNR and LODDS were also reported to be associated with GC prognosis [21–23]. These above studies mainly focused on the relationship between RLN count and long-term prognosis. However, little is known about its effects on postoperative short-term complications.

In this study, we concentrated on the association between RLN count and short-term prognosis. Univariate analyses showed no significant difference between RLN count and postoperative complications (both overall and stratified by CDC grade). Therefore, more lymph nodes were encouraged to be dissected from the perspective of short-term prognosis.

Although curative gastrectomy with D2 lymphadenectomy is considered a pivotal strategy for advanced GC, there are international and institutional differences in the number of RLN count [24, 25]. Various factors were reported to influence the RLN count, including the confidence and enthusiasm of doctors (both surgeons and pathologists), surgical situation, and innate lymph node count in each patient [7, 9]. In our study, we concluded that RLN count was related to the type of resection, tumor invasion, and lymph node metastasis. Of note, RLN count was positively correlated with the lymph node metastasis rate, which underlined the importance of RLN count for accurate staging.

Actually, for a thorough pathological examination, RLNs should be individually divided from a complete tissue sample after surgery. Owing to much time and effort was required during this procedure, it has not been widely implemented clinically. Therefore, the examined lymph node count by pathologists might be lower than the dissected lymph node count. Multiple attempts have been conducted to improve the detection rate of lymph nodes [26–28]. Li et al. elucidated that the mean number of RLNs could be significantly elevated by injecting carbon nanoparticles before surgery compared with controls (38.33 vs 28.27) [26]. Markl and colleagues reported a twofold lymph node pick up rate utilizing methylene blue staining than unstained groups (35 vs 17) [27]. Several dye materials were also used to increase the number of lymph nodes dissected during surgery, such as fluorescent indocyanine green (ICG) and 5-aminolevulinic acid (5-ALA) [29, 30].

We acknowledge that this study had some potential limitations. First, it was a retrospective, single-center study, so the results might be flawed because of residual confounding factors. Second, the RLN count was closely related to the quality of surgeons and pathologists. The perioperative variables might differ in different doctors. Therefore, multicenter studies are needed to confirm our results.

## Conclusions

In conclusion, the current study demonstrated that RLNs\ count was not associated with postoperative short-term complications following gastrectomy of GC. Therefore, our analysis encouraged more lymph nodes to be dissected for accurate pathologic staging.

## Data Availability

Access to the data and the calculation method can be obtained from the authors by email (medsunfeng@163.com).

## Abbreviations

BMI: Body mass index
CRP: C-reactive protein
ASA: American Society of Anesthesiologists
RLNs: Retrieved lymph nodes
LNR: Lymph node ratio
LODDS: Log odds of positive lymph nodes
